# Overexpression of MCM3 as a prognostic biomarker correlated with cell proliferation, cell cycle and immune regulation in hepatocellular carcinoma

**DOI:** 10.7150/jca.104325

**Published:** 2025-01-27

**Authors:** Linling Ju, Huixuan Wang, Yunfeng Luo, Yichen Wang, Lin Chen, Xudong Han, Rujian Lu

**Affiliations:** 1 Medical School of Nantong University, Nantong University, Affiliated Nantong Hospital 3 of Nantong University, Nantong Third People`s Hospital, Nantong 226000, Jiangsu, China.; 2 Ulink High School of Suzhou Industrial Park, Suzhou 215006, Jiangsu, China.

**Keywords:** MCM3, hepatocellular carcinoma, cell cycle, prognosis, immune cell infiltration

## Abstract

**Background:** Hepatocellular carcinoma (HCC) is a common malignant tumor and has a poor prognosis. Minichromosome maintenance 3 (MCM3) protein is upregulated in several cancers, but the biological function, molecular mechanisms and the relationship with tumor immunity of MCM3 in HCC remain poorly understood.

**Methods:** The expression levels and prognosis role of MCM3 in HCC were analyzed based on TCGA, GEO and LIHC databases, and 40 paired tissue samples. We conducted Kyoto Encyclopedia of Genes and Genomes (KEGG) and Gene Ontology (GO) analyses on these DEGs to explore the potential impact of MCM3 on the biological behavior of HCC. In addition, flow cytometry, CCK-8, EdU, colony formation and nude mice xenograft models were employed to investigate the biological functions of MCM3. Furthermore, immune cell infiltration, markers and checkpoint-associated genes were analyzed by TIMER 2.0, ACLBI and TCGA database.

**Results:** In this study, we investigated the expression and function of MCM3 in HCC. MCM3 was highly expressed in a variety of tumors including HCC, and high MCM3 expression was positively associated with various clinicopathological parameters and acted as an independent factor of the poor prognosis for overall survival in HCC. Meanwhile, immune characteristics analysis indicated that high MCM3 expression was related to the level of immune cell infiltration and immune checkpoints in HCC. Our functional enrichment analysis indicated that MCM3 is mainly involved in the cell cycle and cell metabolic related pathways. Moreover, *in vitro* and *in vivo* experiments further confirmed that MCM3 could promote the proliferation of HCC by regulating cell cycle progression.

**Conclusions:** Our results indicated that MCM3 was up-regulated in HCC and might become a biomarker in the diagnosis and treatment of patients with HCC.

## Introduction

Hepatocellular carcinoma (HCC) is one of the leading causes of cancer-related deaths and the sixth most common malignant tumor worldwide^1^. At present, there are many methods for treating liver cancer, including interventional techniques, surgical resection, and liver transplantation^2^. The most effective treatment for HCC patients is surgical resection, but the risk of recurrence within 5 years is as high as 70%. In addition, most HCC patients miss the opportunity for surgical treatment due to the lack of typical clinical manifestations in the early stage^3^, ^4^. In recent years, an increasing number of clinical studies have explored the efficacy of immunotherapy for HCC^5^, ^6^. However, our understanding of HCC immunotherapy is still insufficient. Therefore, there is a need to develop potential biomarkers that accurately predict the prognosis of patients and may serve as targets for immunotherapy.

The progression of HCC was closely related to the dysregulation of genes and signaling pathways^7^. The minichromosome maintenance proteins (MCMs) are mainly located in nucleus of cancer cells and play a critical role in the initiation of the cell division and DNA replication process^8^-^10^. MCM3 belongs to the MCMs family, which consists of eight members, namely MCM2-9. As studies have reported, on the one hand, high expression of MCM3 is associated with poor clinical outcomes of breast cancer^11^, squamous cell carcinomas^12^, renal cell carcinoma^13^ and gastric cancer^14^. On the other hand, MCM3 is not only correlated with poor prognosis but also significantly correlated with immune cell infiltration in cervical cancer^15^, glioma^16^, lung squamous cell carcinoma^17^ and skin cutaneous melanoma^18^. However, currently there are few reports on the function of MCM3 in the progression of HCC. Moreover, in recent years, the role of tumor microenvironment in tumor progression and treatment has gradually become prominent. The results of this study prompt us to explore the interaction mechanism between MCM3 and tumor immunity and the potential function of MCM3 in HCC.

In this study, we first evaluated the relationship between MCM3 expression and prognosis in HCC. Subsequently, functional and pathway enrichment analysis was conducted to explore the potential function of MCM3 in tumor occurrence and development. In addition, we investigated the association of MCM3 expression with immune cell infiltration and immune checkpoints in the HCC microenvironment. Finally, the effects of MCM3 on the cell proliferation and cell cycle were explored *in vitro* and *in vivo* experiments. Our findings provide new insights into the mechanism of MCM3 in HCC and provide new targets for the diagnosis, treatment and prognosis of HCC.

## Materials and Methods

### Datasets sources and ethics statement

TCGA data were download from The Cancer Genome Atlas database (https://portal.gdc.cancer.gov/) and the UCSC Xena database (https://xenabrowser.net/datapages/). Transcriptional profiles of two independent cohorts GSE14520 and GSE45267 that related to HCC were retrieved from GEO datasets (https://www.ncbi.nlm.nih.gov/geo/). We selected 40 paired tissue samples from patients with primary liver cancer. The selection criteria were as follows: Inclusion criteria: (I) HCC was definitively diagnosed by postoperative pathology; (II) the patient's medical records were relatively complete; (III) no comprehensive antitumor treatments, such as radiotherapy, chemotherapy, targeted therapy, or immunotherapy, were per formed before the operation. Exclusion criteria: (I) extrahepatic malignancies and/or non-invasive liver conditions; (II) hepatitis or cirrhosis caused by other diseases, and (III) other diseases that could affect the study outcomes. The samples were acquired from the Nantong Third People's Hospital Affiliated with Nantong University and the study was approved by the Ethics Committee of Nantong Third People's Hospital and written informed consent was obtained from each participant. All samples were frozen immediately after collection and stored at -80˚C until further use.

### Analysis of MCM3 expression in HCC

We explored the expression levels of MCM3 in pan-cancer using the TIMER 2.0 database (http://timer.comp-genomics.org) and Sangerbox platform (http://SangerBox.com/Tool). In addition, we used gene expression data from the HCCDB database (http://lifeome.net/ database/hccdb) to analyze the expression of MCM3 in tumor and normal samples of HCC.

### Survival analysis of MCM3 in HCC

Kaplan-Meier plot database (http://kmplot.com/analysis/) was used to analyze the survival differences between high and low MCM3 expression groups. According to the optimal cutoff value, the patients were divided into MCM3 high and low expression cohorts, and overall survival (OS), first progression (PFS), and disease-specific survival (DSS) curves were plotted. In addition, univariate and multivariate Cox regression analyses were performed to assess the correlation of MCM3 expression with overall survival (OS) and other clinical characteristics in order to determine the prognostic factors of the HCC patients. To evaluate the predictive value of MCM3 for OS in HCC patients, we constructed ROCs using the R package "timeROC" and SPSS 26.0 sofware was used for data analysis.

### Functional enrichment analysis

Gene ontology (GO) and Kyoto Encyclopedia of Genes and Genomes (KEGG) analysis was performed using the “clusterProfiler” R packages, and the results were plotted by “ggplot2” R package. Significant enrichment was considered eligible if *p* < 0.05. GSEA is a computational method used to determine whether a set of prior defined genes exhibit statistical significance between two biological states. GSEA was performed by GSEA version 4.1.0 software. FDR < 0.25, NOM p-value < 0.05 and |NES|> 1 were considered significant enrichment.

### Analysis of immune infiltration

We used two different online platforms, the ACLBI (https://www.aclbi.com/static/index.html#/) and TIMER 2.0 database (http://timer.comp-genomics.org) to analyze the relationships between the tumor immune cell and MCM3 expression. We analyzed the TCGA-LIHC cohort and the UCSC Xena database to illuminated the correlation between MCM3 and seven immune checkpoints by using the Sangerbox platform and SPSS 26.0 software.

### Reverse transcription quantitative polymerase chain reaction (RT‒qPCR)

Total RNA was extracted from tissue or cell samples by TRIzol reagents (Invitrogen, Carlsbad, CA, USA). RNA was reverse-transcribed using PrimeScript-RT-Master Mix (Takara, Beijing, China), cDNA was subsequently used as template, and MCM3 expression was detected by SYBR Green (Vazyme, Nanjing, China). GAPDH was used as the internal control gene. Primer sequences are listed in Table 1.

### Western blot analysis

Total proteins were isolated from cells and HCC tissues using RIPA lysis buffer (Beyotime, Shanghai, China) with protease and phosphatase inhibitors. The proteins were separated by 10% SDS-PAGE and transferred onto the nitrocellulose membranes (Millipore Corporation, USA). The membranes were blocked in rapid block buffer (Beyotime, Shanghai, China) for 15 min and then incubated with primary antibodies at 4 ˚C overnight. Following this, the Horse Radish Peroxidase (HRP)-conjugated secondary antibody incubated the membranes at room temperature for 1 h. The antibodies used in western blotting were as follows: HRP-conjugated-β-actin (Proteintech, China, 1:5000 dilution), MCM3 (Proteintech, China, 1:2000 dilution), CyclinD1 (Proteintech, China, 1:2000 dilution), CyclinE1 (Proteintech, China, 1:2000 dilution), CDK2 (Proteintech, China, 1:2000 dilution), p21 (Proteintech, China, 1:2000 dilution), PCNA (Santa Cruz, Dallas, 1:200 dilution) and HRP-conjugated goat anti-rabbit antibody (Proteintech, China, 1:5000 dilution). The protein bands were visualized by ECL detection system (Tanon, China) then analyzed by Image Studio software and normalized to internal control β-actin.

### Immunohistochemistry (IHC) staining

Forty HCC tissues and adjacent tissues were acquired from the Nantong Third People's Hospital Affiliated with Nantong University. The paraffin sections (4-μm-thick) were dewaxed and dehydrated. For IHC, paraffin sections were incubated overnight with MCM3 or PCNA primary antibodies (1:200) at 4 ˚C. Next, the paraffin sections were washed with PBS and incubated at room temperature with HRP-conjugated secondary antibody (Santa Cruz) for 1 h. Finally, the paraffin sections were stained with DAB and hematoxylin. The IHC sections were further detected under a microscope (Olympus, Japan). The expression level of MCM3 was evaluated using ImageJ software, and the semi-quantitative scoring was categorized as strongly positive (4), moderately positive (3), weakly positive (2), and negative (1) staining.

### Cell culture and cell cycle analysis

The PLC/PRF/5, SK-HEP-1, Li-7 and the normal human liver LO2 cell lines were purchased from the Chinese Academy of Sciences (Shanghai, China). The PLC/PRF/5 and SK-HEP-1 cell lines were cultured in MEM (Gibco), while the other two cell lines were cultured in DMEM (Gibco). All the cells were cultured in medium supplied with 10% (v/v) fetal bovine serum (FBS, Gibco) in a humidified incubator (37 ˚C, 5% CO_2_). Cell cycle analysis was evaluated using the cell cycle kit (DOJINDO Laboratories, Japan) in accordance with the manufacturer's protocol. The results were measured by a FACSCalibur flow cytometer (BD Biosciences) and analyzed by FlowJo software 10.7.

### CCK-8 and colony formation assay

Cell proliferation was evaluated using the CCK-8 kit (DOJINDO Laboratories, Japan) in accordance with the manufacturer's protocol. Briefly, 10 μL of CCK8 solution was added to the cells, followed by incubation for 2 h at 37 ℃. The absorbance at 450 nm was then measured using a microplate reader. 1×10^3^ transfected cells were added to 6-well plates. After two weeks the cell colonies were fixed with 4% paraformaldehyde (Sangon, Shanghai, China) and stained with 0.1% crystal violet (Beyotime, Shanghai, China).

### 5-Ethynyl-20-deoxyuridine (EdU) assay

The EdU assay was carried out with a Cell-Light EdU DNA Cell Proliferation Kit (RiboBio, Guangzhou, China) in accordance with the manufacturer's protocol. All images were acquired with an Olympus IX73-FL-PH fluorescence microscope (Olympus, Tokyo, Japan).

### Animal experiments

Five-week-old female BALB/c nude mice (n=6) for tumor xenografts experiments were purchased from Nantong University. PLC/PRF/5 cells stably transfected with shRNA plasmids or control vector were subcutaneously injected into the upper back of the nude mice (1 × 10^7^, 100 μL). One month after injection, mice were sacrificed and detected for tumor weight, gene expression. All procedures were approved by the Animal Care Committee of Nantong University.

### Statistical analysis

The experimental data were analyzed using GraphPad Prism 7.0 (GraphPad, CA, USA) and SPSS version 17.0 software. The student's t-test was used for comparison between two groups. The differences among three or more groups were analyzed by the one-way analysis of variance (ANOVA). Survival curve was constructed with the Kaplan-Meier method and log-rank test. P <0.05 was considered statistically significant.

## Results

### The MCM3 expression in pan-cancer

**Figure 1:**
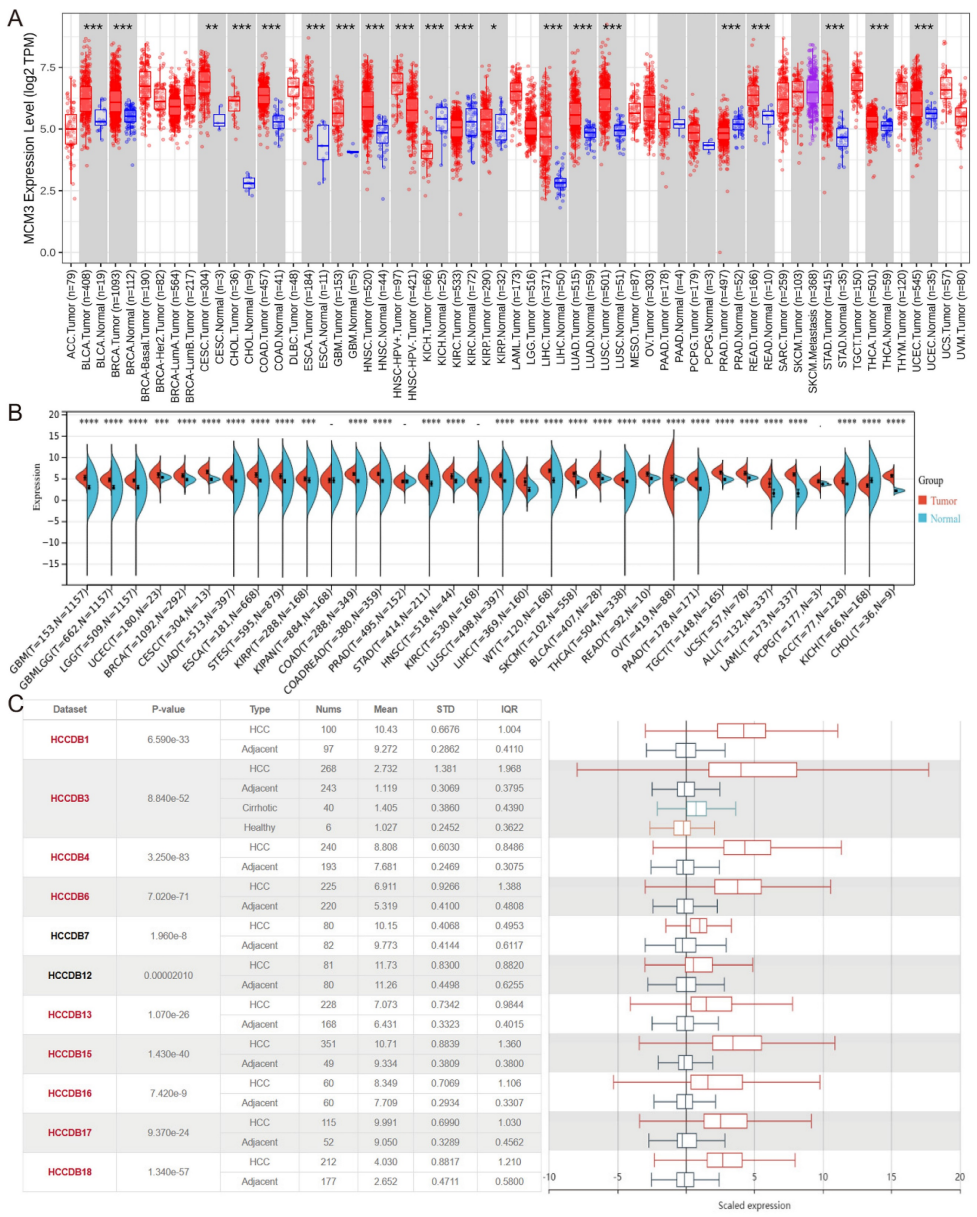
Database analysis of MCM3 expression in HCC. (**A-C**) Pan-cancer expression analysis of MCM3 between normal and tumor samples according to TIMER 2.0, Sanger box and HCCDB database.

To compare the expression of MCM3 between tumor and non-tumor tissues, we performed a pan-cancer expression analysis of MCM3 at TIMER 2.0 database. The results showed that MCM3 was significantly overexpressed in cholangiocarcinoma (CHOL), Colon adenocarcinoma (COAD), esophageal carcinoma (ESCA), LIHC, Lung adenocarcinoma (LUAD), lung squamous cell carcinoma (LUSC), rectum adenocarcinoma (READ), stomach adenocarcinoma (STAD) and other cancers (Figure 1A). Next, data in Sangerbox (Figure 1B) and HCCDB (Figure 1C) confirmed that MCM3 is highly expressed in HCC.

### Association of MCM3 expression and clinicopathological characteristics in HCC

**Figure 2:**
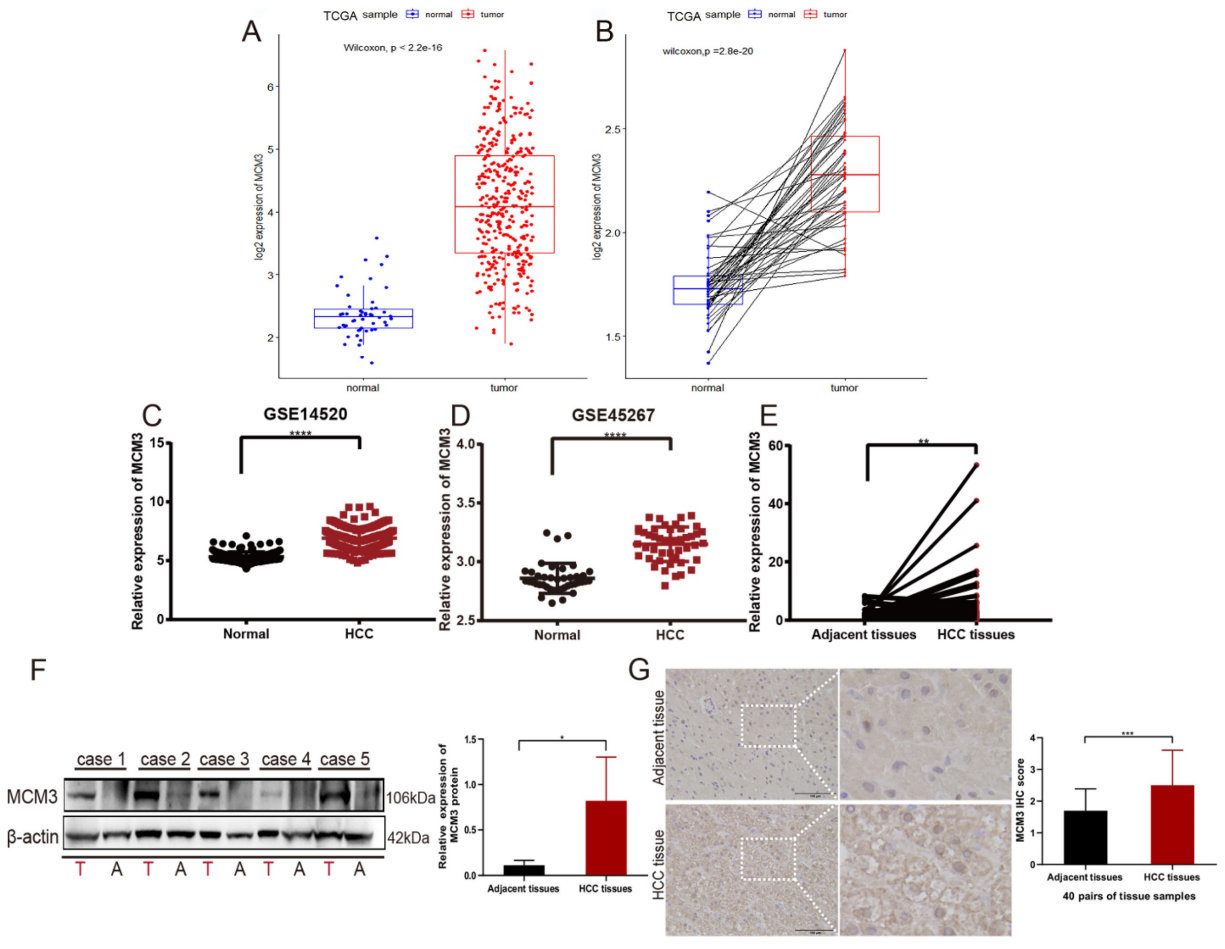
Increased MCM3 expression in HCC tissues. (**A-D**) The expression of MCM3 was elevated in HCC samples in the TCGA, GSE14520 and GSE45267 databases. (**E-G**) The mRNA and protein levels of MCM3 in HCC tissues compared to adjacent tissues, as detected by qRT-PCR, Western blot and IHC. All experiments were performed in triplicate.

To further explore the association between MCM3 expression and HCC patients, we compared MCM3 expression to that in normal tissues. The results showed that the expression of MCM3 was significantly increased in tumor tissues (P < 0.001) (Figure 2A). This finding was confirmed in tumor tissues and paired normal tissues (P < 0.001) (Figure 2B). Furthermore, we used GEO database and also found that MCM3 expression was elevated in HCC (Figures 2C and D). We confirmed by qRT-PCR and Western blotting that MCM3 mRNA and protein levels were both upregulated in HCC tumor tissues compared with that in paired normal adjacent tissues (Figures 2E and F). Consistently, IHC staining of MCM3 showed similar increased in HCC tissues (Figure 2G). In addition, we explored the clinicopathological factors of HCC patients in the different MCM3 expression groups. It was found that the MCM3^high^ group was significantly correlated with T classification, M classification and Tumor stage (Table 2).

### The predictive value of MCM3 in the early diagnosis and prognosis of HCC

**Figure 3:**
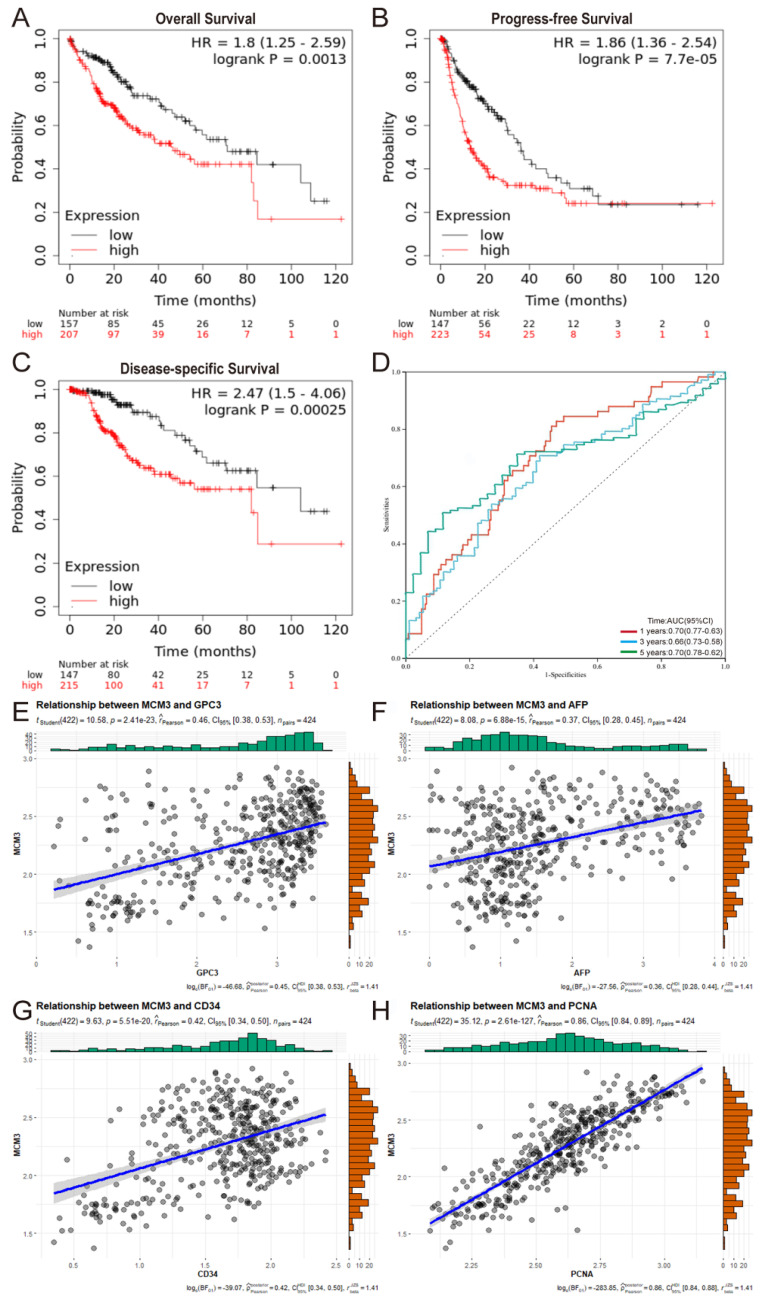
Prognostic and diagnostic value of MCM3 in HCC. (**A-C**) Kaplan-Meier curves of OS, PFS, and DSS. (**D**) Time-dependent ROC curves of risk score model at 1-, 3-, and 5-years- overall survival of HCC in the training group. (**E-H**) Correlation analysis between MCM3 and GPC3, AFP, CD34, PCNA based on TCGA database.

Then, Kaplan‒Meier survival curves were used to evaluate the association between MCM3 expression and the survival outcomes. The results indicated that the OS, PFS, and DSS rates of HCC patients in the MCM3^high^ group was significantly lower than those with MCM3^low^ (Figures 3A-C). Collectively, these findings suggest that MCM3 has good prognostic value in HCC. In addition, we plotted ROC curves to evaluate the predictive accuracy and risk scores of MCM3 in HCC patient survival analyses (Figure 3D). The areas under the curve (AUCs) for 1-, 3-, and 5-years were 0.70, 0.66, and 0.70, respectively, indicating that MCM3 had a good ability to predict the prognosis of HCC patients. Furthermore, we performed univariate and multivariate analyses to identify survival predictors to confirm that MCM3 was an independent prognostic factor for HCC survival. Univariate Cox regression analysis showed that T classification (p < 0.001, HR = 1.633, 95% CI: 1.345-1.983), M classification (p = 0.046, HR = 1.516, 95% CI: 1.008-2.279), tumor stage (p < 0.001, HR = 1.652, 95% CI: 1.348-2.026) and MCM3 expression (p = 0.007, HR = 1.668, 95% CI: 1.151-2.418) were significantly associated with the prognosis of HCC patients. The results of multivariate Cox regression analysis showed that MCM3 expression (p = 0.014, HR = 1.601, 95% CI: 1.098-2.335) was independent risk factor affecting the prognosis of HCC patients. In conclusion, these results implied that MCM3 could serve as a potential prognostic indicator for HCC.

Alpha-fetoprotein (AFP) as a more sensitive and specific biochemical marker of primary hepatic carcinoma (PHC). However, many HCC patients show normal serum AFP levels in the early stages**19**. Therefore, it is necessary to develop reliable biomarkers for early diagnosis of HCC. Based on TCGA database analysis, MCM3 was strongly correlated with glypican-3 (GPC3), AFP, CD34 and proliferating cell nuclear antigen (PCNA) expression in HCC. These proteins are closely related to the diagnosis of HCC and tumor proliferation (Figures 3E-H). In summary, MCM3 has good prognostic and diagnostic value in HCC.

### Effect of MCM3 on the biological functions of HCC

**Figure 4:**
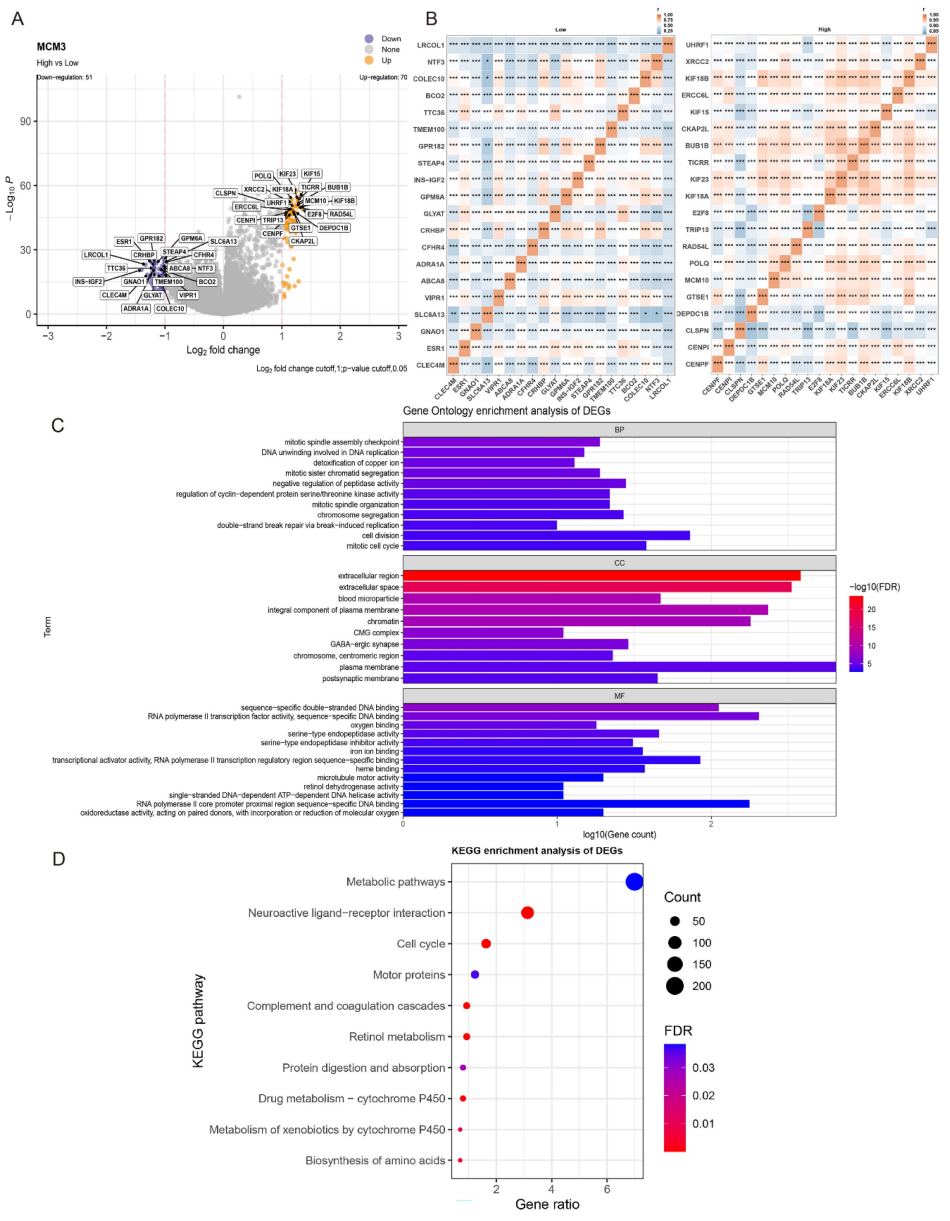
Risk score-related enrichment analysis in HCC patients. (**A, B**) The results of difference analysis between the high- and low-groups presented by volcano plot and heat map. (**C, D**) GO and KEGG Pathway Analysis results for MCM3 in HCC.

To further explore the potential function of MCM3 in tumor occurrence and development, we evaluated DEG in the TCGA-HCC cohort between the MCM3^high^ and MCM3^low^ groups. The DEGs results included statistically significant 70 up-regulated genes and 51 down-regulated genes (Figure 4A). The heat map of the relative expression values of the first 20 DEGs between the high and low MCM3 groups is also shown in Figure 4B. We used Gene Ontology (GO) and Kyoto Encyclopedia of Genes and Genomes (KEGG) enrichment analyses to explore the functional mechanism of MCM3 in HCC development. We discovered these DEGs were predominantly enriched in cell cycle and cell metabolic related biological process (BP) terms “mitotic cell cycle”, “cell division”, “regulation of cyclin-dependent protein serine/threonine kinase activity” and “detoxification of copper ion”. The significantly enriched cellular component (CC) terms “chromatin”, “chromosome centrmeric region”, “CMG complex”; the molecular function (MF) terms “RNA polymerase II transcription factor activity”, “serine-type endopeptidase activity”, “iron ion binding” “RNA polymerase II core promoter proximal region sequence” and “oxidoreductase activity” (Figure 4C). KEGG analysis proved that they were implicated in “Metabolic pathways” and “Cell cycle” (Figure 4D). The results of the GO and KEGG analysis showed MCM3 and its co-expressed DEGs were enriched in cell cycle and cell metabolic related pathways, suggesting that MCM3 was critically involved in these processes.

### MCM3 promotes HCC cells proliferation *in vitro*

**Figure 5:**
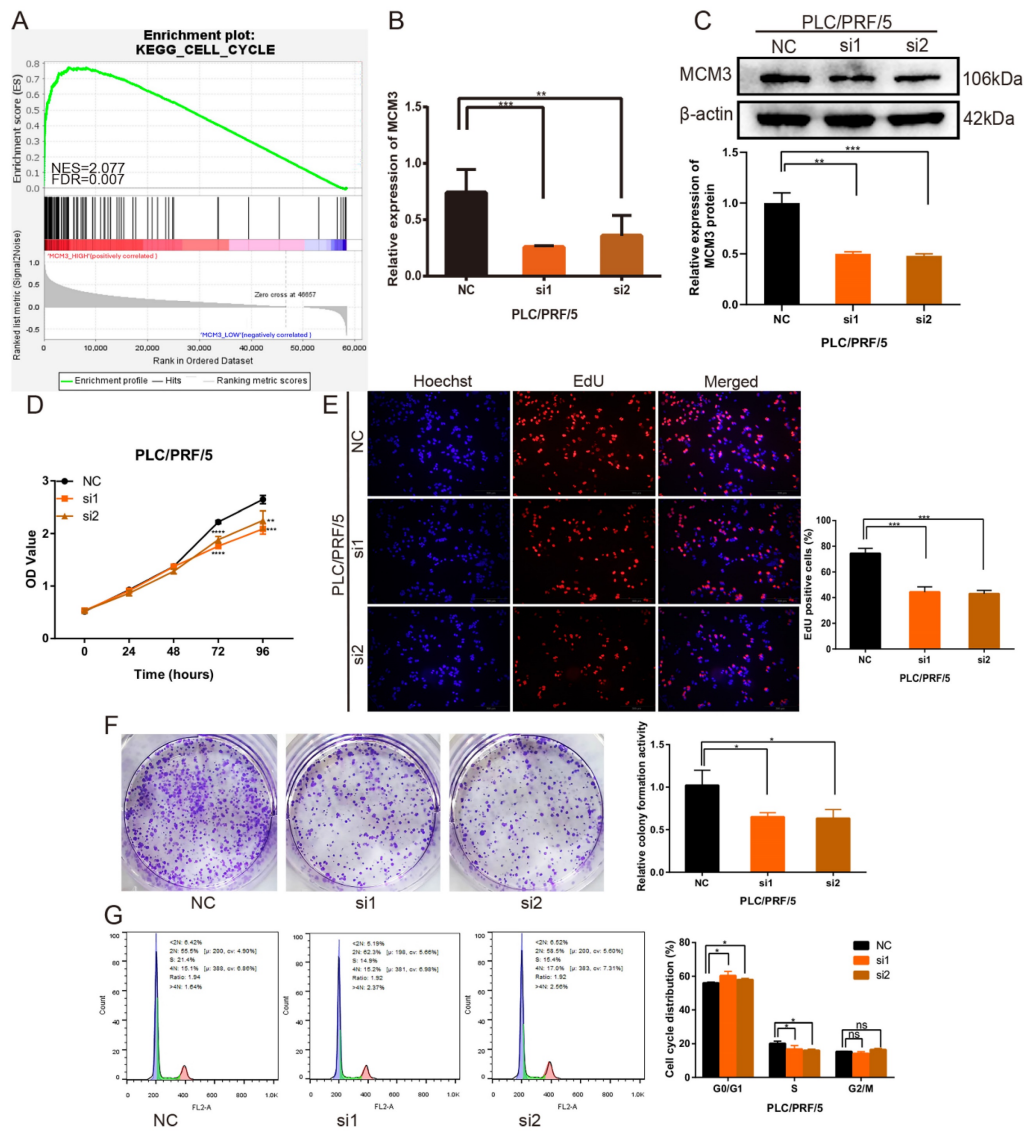
Downregulation of MCM3 inhibits HCC cells proliferation *in vitro*. (**A**) GSEA plot showing that MCM3 expression is positively correlated with cell cycle in HCC. (**B, C**) Small interference mediated MCM3 downregulation was determined by qRT-PCR and western blotting in PLC/PRF/5 cells. (**D**) CCK-8 assay showed the cell growth ability. (**E**) The EdU assay was used to detect cell ability of proliferation induced by downregulation of MCM3. (**F**) Colony formation assay indicated the cell growth ability. (**G**) Cell cycle distribution was assessed by flow cytometry analysis. All experiments were performed in triplicate. Statistical significance was assessed by two-tailed Student's t test. The error bar indicates the SD. *P<0.05; **P<0.01; ***P<0.001; ****P<0.0001.

To verify the important role of MCM3 in HCC progression the potential related mechanisms, we used the GSEA analysis to analyze the gene sets with altered MCM3 expression. The results found MCM3 was positively associated with cell cycle progression (Figure 5A). Specific siRNA targeting MCM3 were transfected into PLC/PRF/5 cells to investigated the effect of MCM3 on the growth of HCC cells. After transfected with MCM3-siRNA, the mRNA and protein expression of MCM3 was downregulated significantly in PLC/PRF/5 cells (Figures 5B and C). Cytoplasma-targeting CCK8 assay and nucleus-targeting EdU assay were performed to detect cell proliferation ability. Both assay results showed that downregulation of MCM3 significantly suppressed the growth ability of PLC/PRF/5 cells (Figures 5D and E). Similarly, colony formation assay demonstrated that the frequency of foci formation in cells decreased with the downregulation of MCM3 (Figure 5F). Next, we used flow cytometry to detect the effect of MCM3 on the cell cycle to evaluate whether MCM3 promoted cell growth and proliferation through cell cycle arrest. The results suggested that cell cycle assay showed that MCM3 depletion significantly decreased the percentage of PLC/PRF/5 cells in the G0/G1 phase (Figure 5G).

**Figure 6:**
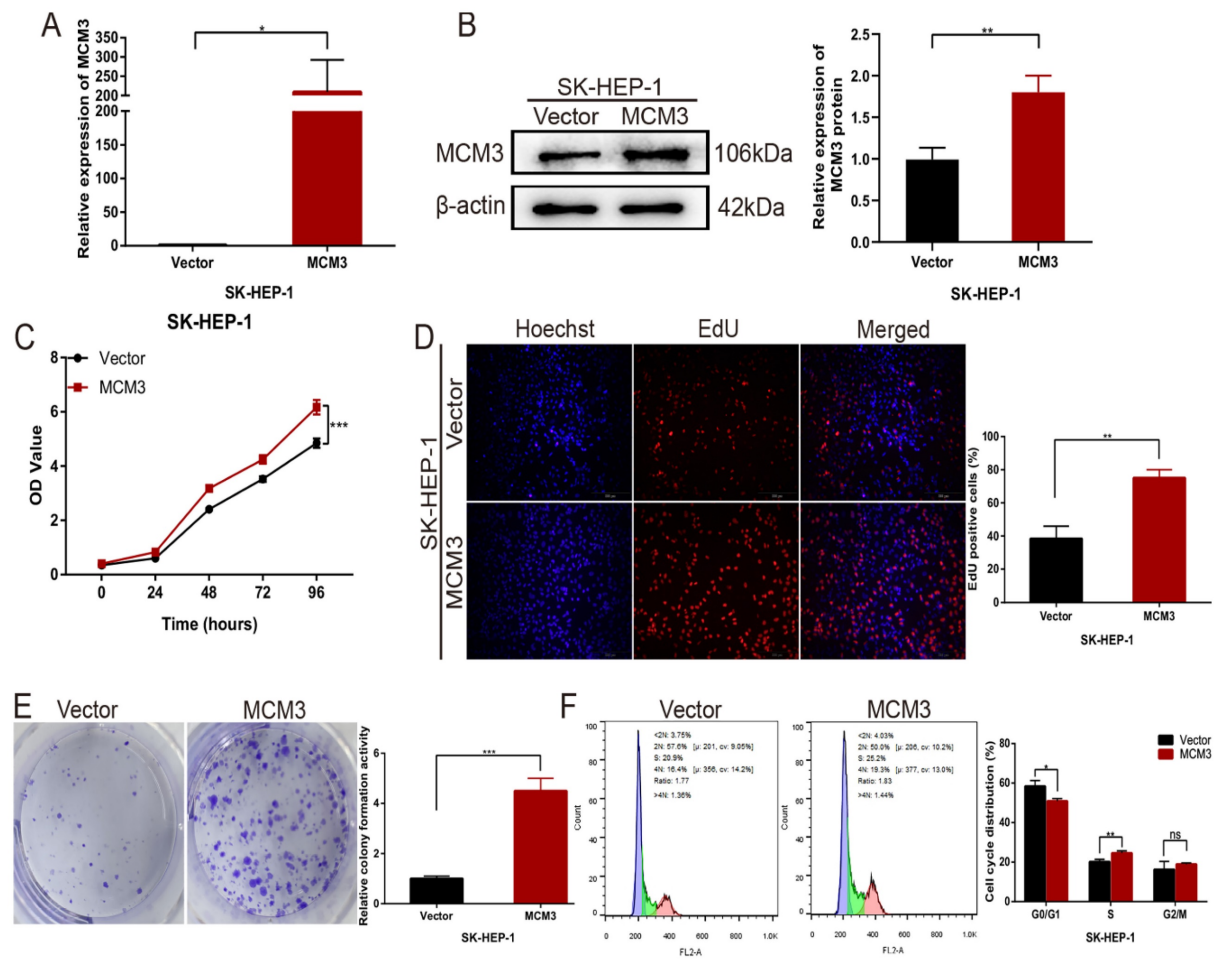
Upregulation of MCM3 promotes HCC cells proliferation *in vitro*. (**A**) The efficiency of MCM3 overexpression was confirmed by qRT-PCR in SK-HEP-1 cells. (**B**) Western blotting analysis of overexpression efficiency of MCM3 in SK-HEP-1 cells. (**C**) CCK-8 assay showed the cell growth ability. (**D**) The EdU assay was used to detect cell ability of proliferation induced by overexpression of MCM3. (**E**) Colony formation assay indicated the cell growth ability. (**F**) Cell cycle distribution was assessed by flow cytometry analysis. All experiments were performed in triplicate. Statistical significance was assessed by two-tailed Student's t test. The error bar indicates the SD. *P<0.05; **P<0.01; ***P<0.001.

Next, we overexpressed MCM3 in cells to explore the function of MCM3 in HCC cell proliferation. Stable MCM3-overexpressing cell line was established using SK-HEP-1 cells. The mRNA and protein expression of MCM3 was upregulated significantly in SK-HEP-1 cells (Figures 6A and B). The CCK-8 assay and EdU assay indicated that cells with MCM3 overexpression exhibited promotion of growth relative to cells in the empty vector group (Figures 6C and D). Consistent with the cell proliferation result, colony formation assay displayed that overexpression of MCM3 increased the cell colonies compared to vector group (Figure 6E). We also evaluated the effect of MCM3 on cell cycle distribution to elucidate the potential mechanism of MCM3 on HCC cell proliferation. The results showed that MCM3 overexpression significantly induced SK-HEP-1 cells arrest in the G0/G1 phase (Figure 6F). These suggested that MCM3 played an essential role in the proliferation of HCC cells.

### MCM3 as a cell cycle regulator promotes the proliferation of HCC

**Figure 7:**
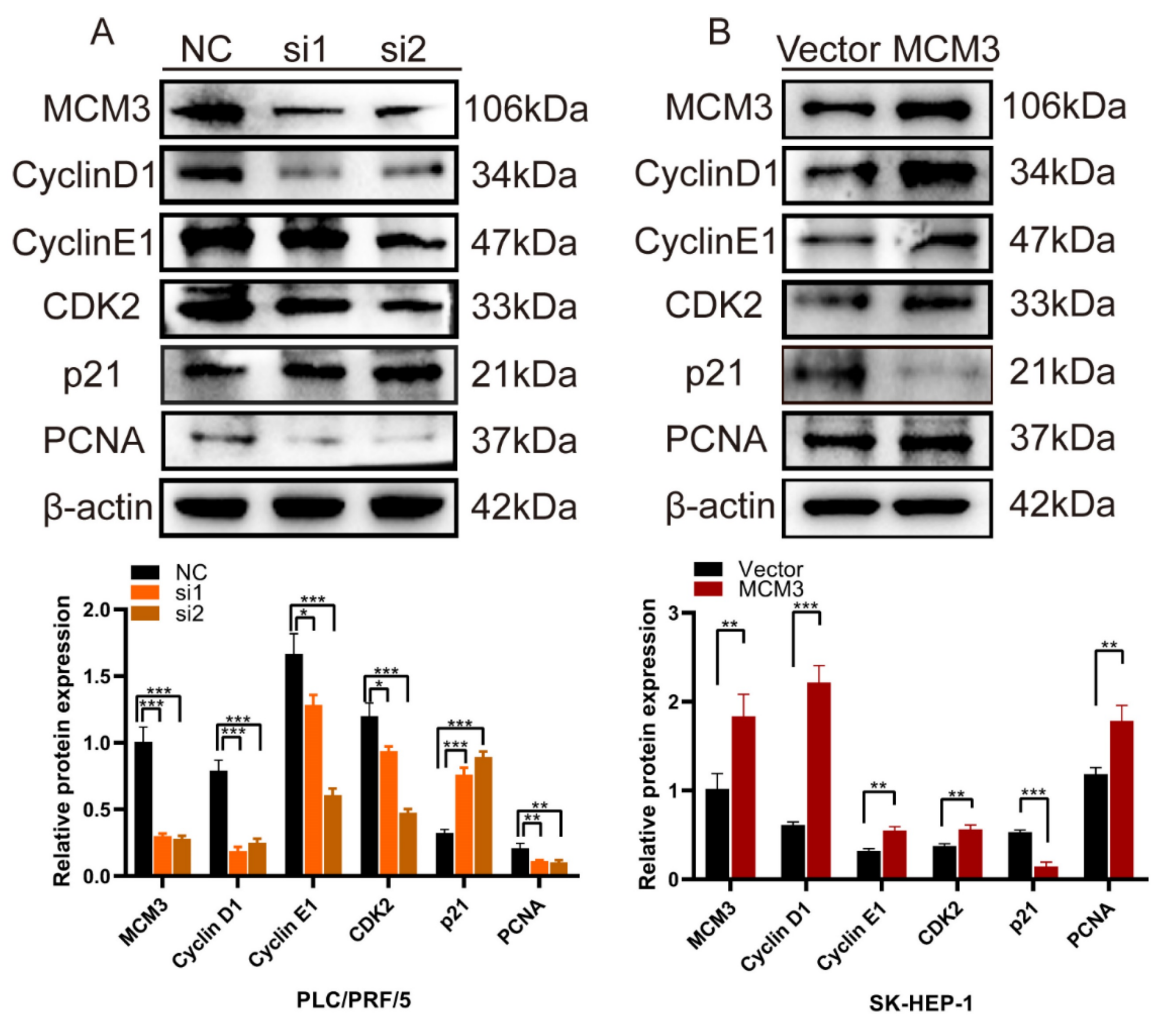
Knockdown and overexpression of MCM3 affects cell cycle and proliferation related proteins. (**A, B**) Western blotting results demonstrated that the knockdown or overexpression of MCM3 influenced the expression levels of the Cyclin D1, Cyclin E1, CDK2, p21 and PCNA. Analysis of the expression levels of related proteins in PLC/PRF/5 and SK-HEP-1 cells. All experiments were performed in triplicate.

We assessed the effects of MCM3 downregulation or overexpression on the expression of cell cycle and proliferation related proteins, including Cyclin D1, Cyclin E1, CDK2, p21 and PCNA. We noticed that the expression levels of Cyclin D1, Cyclin E1, CDK2 and PCNA were significantly inhibited and P21 was increased when expression of MCM3 was downregulated (Figure 7A). However, MCM3 overexpression led to the opposite results (Figure 7B). All these results indicated that MCM3 acted as a cell cycle regulator to promote the proliferation of HCC.

### Downregulation of MCM3 represses the tumor growth *in vivo*

**Figure 8:**
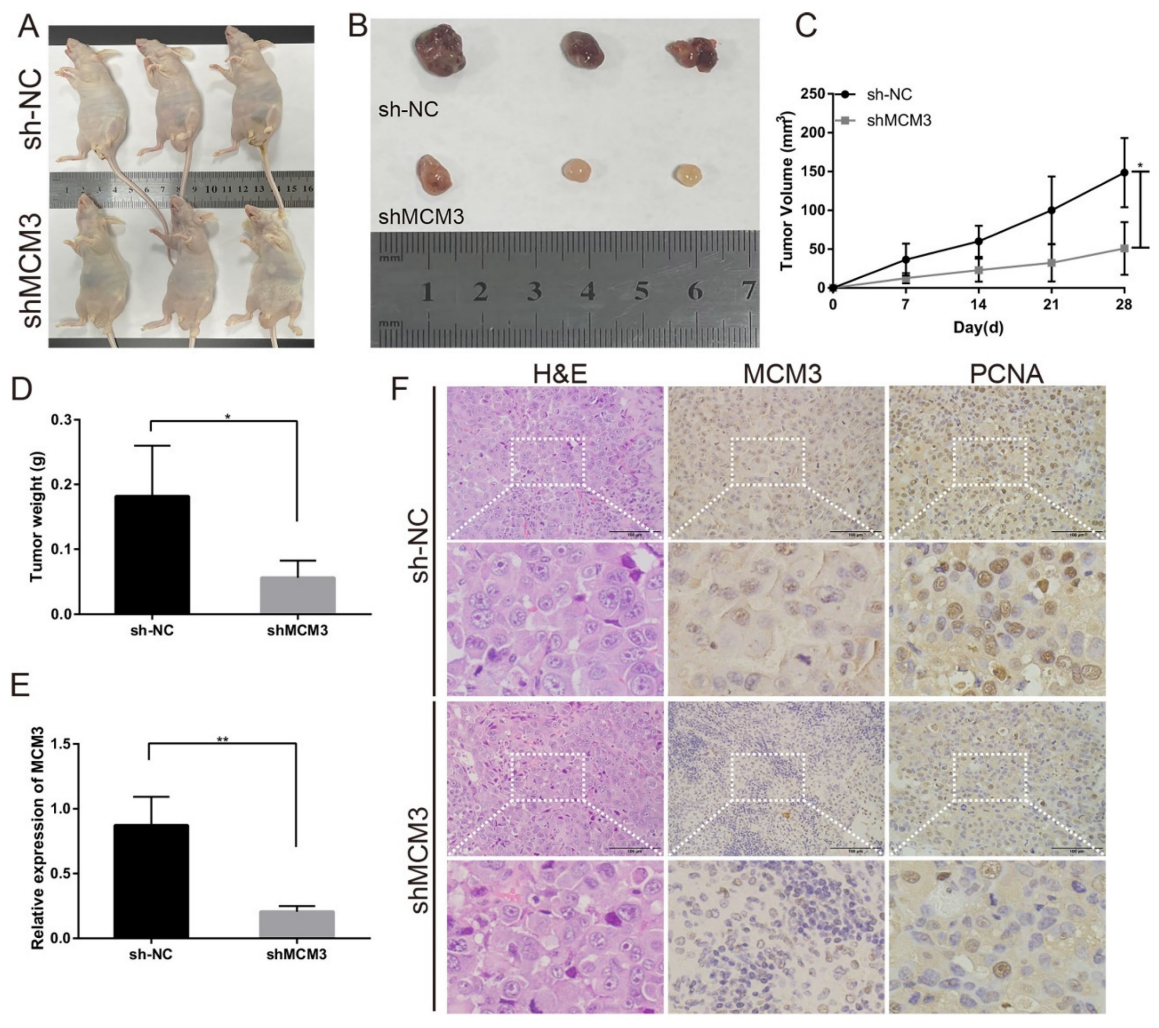
Effect of MCM3 on tumor growth *in vivo*. (**A**) Image of nude mice injected with PLC/PRF/5 cells subcutaneously (1×10^7^ cells per mouse, n=3 for each group). (**B**) Tumor collected from mice were measured after one month of hypodermic injection. (**C, D**) Tumor volumes and weights were measured. (**E**) The relative expression levels of MCM3 in tumors were determined by qRT-PCR analysis. (**F**) H&E staining showed the structure of tumors. Immunohistochemical staining of MCM3 and PCNA expression in tumors. Scale bar, 100μm. Statistical significance was assessed by two-tailed Student's t test. The error bar indicates the SD. *P<0.05; **P<0.01.

To investigate the effect of MCM3 on tumor growth *in vivo*, PLC/PRF/5 cells transfected with shNC or shMCM3 were subcutaneously injected into nude mice. Tumors in nude mice were markedly smaller in the shMCM3 group compared with those in the shNC group (Figures 8A and B). Consistently, tumor volumes and weights were significantly lower in the shMCM3 group (Figures 8C and D). qRT-PCR analysis demonstrated that the expressions of MCM3 was significantly downregulated in shMCM3 group tumor samples compared with shNC group (Figure 8E). The results of H&E and IHC showed that the positive rate of MCM3 and PCNA was markedly reduced in shMCM3 group tumor samples compared with shNC group (Figure 8F). Taken together, these findings provide evidences that MCM3 regulates cell cycle progression contribute to the proliferation of HCC.

### Correlation between MCM3 expression and immune characteristics

**Figure 9:**
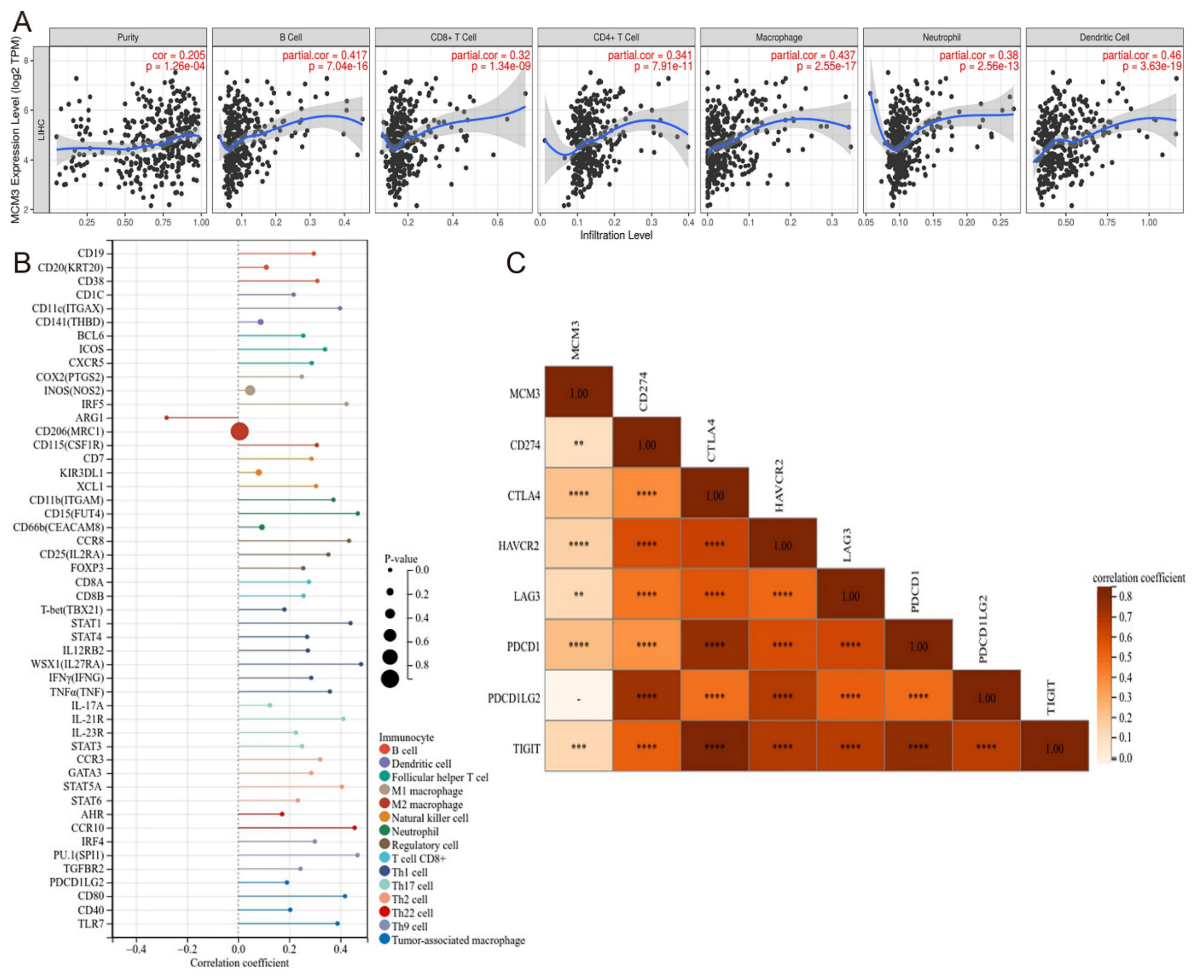
Correlation between MCM3 and immune infiltration in HCC. (**A**) The relationship of MCM3 expression with tumor purity and the infiltration of major immune cells including B cells, CD8^+^ T cells, CD4^+^ T cells, macrophage, neutrophil and dendritic cell from TIMER. (**B**) The forest plot showed the correlation between MCM3 expression and immune cell markers in HCC. (**C**) MCM3 was co-expressed with several immune checkpoints.

Studies have shown that the infiltrating immune cells in the tumor microenvironment are involved in the occurrence, progression, and treatment of HCC^20^. Therefore, we further investigated the correlation between infiltrated immune cells and MCM3 by TIMER. The expression levels of MCM3 and the infiltration levels of B cells, CD8^+^T cells, CD4^+^T cells, macrophage, neutrophil and dendritic cell were significantly positively correlated (Figure 9A). Meanwhile, we found the expression level of MCM3 was significantly correlated with immune cell markers (Figure 9B). The results showed that there were 50 immune cell markers co-expressed with MCM3, and 48 markers showed a positive association. Next, we explored the correlation between MCM3 and immune checkpoints. Among these co-expressed genes, the significantly correlation genes were as follows: Cytotoxic T-Lymphocyte Associated Protein 4 (CTLA4), Hepatitis A Virus Cellular Receptor 2 (HAVCR2), Programmed Cell Death 1 (PDCD1), T Cell Immunoreceptor With Ig And ITIM Domains (TIGIT), CD274 and Lymphocyte Activation Gene-3 (LAG3) all of which were positively correlated (Figure 9C). These results reflected that MCM3 can be widely expressed in immune cells of HCC tumor tissues. Therefore, targeting MCM3 may be a promising method for improving the immune response to tumors.

## Discussion

In the majority of HCC cases, patients are diagnosed at an advanced stage thus missing the optimal time for curative surgery. Therefore, it is necessary to develop relevant biomarkers that can be used for early diagnosis of HCC and accurate prediction of HCC survival prognosis^21^. MCM3 has been shown to be a good surrogate marker of proliferation in tumors from other sites, such as breast carcinoma^22^, colorectal cancer^23^ and oral squamous cell carcinoma^24^. Previous studies evaluating the role of MCM3 in various cancers showed that high expression of MCM3 is associated with poor prognosis, poor clinical outcomes and immune cell infiltration^11^, ^12^, ^17^. In this study, we determined the diagnostic and prognostic value of MCM3 in HCC, as well as its biological function in the development of HCC. In addition, MCM3 participates in the immune infiltration of HCC and is associated with the expression of immune checkpoint genes, laying the foundation for a new method of HCC immunotherapy.

In this study, we found that MCM3 expression was higher in HCC tissues compared to normal tissues in TCGA, GEO and LIHC database analyses. Meanwhile, the mRNA and protein levels of MCM3 were significantly overexpressed in HCC through qRT-PCR, Western Blot and IHC. Next, we explored the correlation between MCM3 and clinical characteristics of HCC patients through the TCGA database. The high expression of MCM3 in HCC patients is associated with T classification, M classification and Tumor stage. These findings suggested that abnormal expression of MCM3 was associated with poor clinical characteristics. Furthermore, we discovered that abnormally high expression of MCM3 was associated with poor OS, PFS and DSS. It indicated that high expression of MCM3 was associated with poor prognosis in HCC patients. Additionally, we found that MCM3 was an independent prognostic factor for HCC through the univariate and multivariate Cox analysis. We further predicted the 1-year, 3-year, and 5-year OS of HCC patients, and the results showed that MCM3 had a good ability to predict the prognosis of HCC patients. Overall, MCM3 had the potential to be a prognostic biomarker for HCC.

Currently, although AFP is one of the most widely used biomarkers for HCC^25^, it has been reported that glypian-3 (GPC3) is superior to AFP in early detection of HCC^26^. CD34 is reported to be the most widely used marker in IHC^27^. PCNA is a kind of serum protein that was speculated associating with AFP negative expression in HCC patient^28^. Therefore, we explored the association between MCM3 expression and these three biomarkers, and showed a significant relationship between MCM3 and three biomarkers. Taken together, MCM3 performed well in the diagnosis and prognosis of HCC patients.

To understand the mechanism by which MCM3 promotes hepatocellular carcinoma, we screened the TCGA dataset and performed GO and KEGG analyses on these DEGs. GO analysis results showed that DEGs were mainly involved in cell cycle and cell metabolic, which could affect division and growth of cancer cells and may even affect the progression of HCC. Similarly, KEGG analysis demonstrated that these DEGs regulate “Metabolic pathways” and “Cell cycle”. These results provide new evidence for the possible involvement of MCM3 in regulating the malignant proliferation and progression of HCC, as well as new insights into the mechanism of MCM3 in HCC. Furthermore, we validated the impact of MCM3 in HCC through a series of experiments *in vitro* and *vivo*. We found that MCM3 promoted the proliferation of HCC cells by blocking the G1/S phase transition. Then, the results of CCK8 and EdU analysis showed that overexpression of MCM3 significantly promoted the growth of HCC cells. Moreover, colony formation assay displayed that overexpression of MCM3 increased the cell colonies compared to vector group. *In vivo* study further confirmed that MCM3 downregulation repressed the tumor growth of HCC cells.

The cell cycle is important in regulating cell proliferation. The complex cyclin-dependent kinases and cyclins can drive cell from one stage to another during cell proliferation^29^, ^30^. The cyclin D1/CDK4 and cyclin E1/CDK2 complexes are essential for promoting cell cycle progression from the G0/G1 to S phases^31^. P21, a CDK inhibitor, regulates the negative feedback of G1/S transition, and increased expression of P21 blocks the progress of G1/S transition^32^-^34^. PCNA protein is a molecular marker of proliferation that is expressed in the early G0/G1 and S phases of the cell cycle. We assessed the effects of MCM3 downregulation or overexpression on the expression of cell cycle- and proliferation-related proteins by western blot analysis. MCM3 downregulation markedly reduced expression of Cyclin D1, Cyclin E1, CDK2 and PCNA, while when MCM3 overexpression plasmid was transfected, the expression of these proteins was all increased.

The tumor microenvironment is a complex ecosystem involved in the occurrence and metastasis of cancer^35^. The tumor immune environment (TIME) is generated by the coexistence and interaction of tumor microenvironment with various immune cells and their products, and its dysfunction can lead to immune evasion through defective antigen recognition or immunosuppressive TME, thus promoting tumor progression, recurrence and drug resistance^36^, ^37^.

Immune infiltrating immune cells (TIICs) can modulate the development and progression of tumors^38^. Our study explored the role of MCM3 in tumor immunity for the first time. Firstly, we investigated the immune infiltration of MCM3 in liver cancer. The findings showed that MCM3 was associated with various immune cells in HCC, including B cells, CD4^+^ T cells, CD8^+^ T cells, neutrophils, macrophages, and dendritic cells (positively correlated with MCM3). Relevant studies have shown that cytotoxic CD8^+^ T cells, CD4^+^ T cells, and NK cells work together to maintain immune surveillance. Meanwhile, the abundant immune cells residing in HCC contribute to immune evasion to promote tumor progression, such as regulatory T (Treg) cells and tumor associated macrophages (TAMs)^39^. TAMs are one of the most abundant types of tumor-infiltrating immune cells, divided into two polarizing phenotypes: tumor suppressive M1 and oncogenic M2^40^. Another important finding in the current study is that macrophage overexpression is strongly associated with poor prognostic survival, and contribute to tumor cell escape into the circulatory system, suppress antitumor immune mechanisms^41^. Similar to TAMs, tumor associated neutrophils (TANs) can be classified as antitumor N1 and protumor N2, which are engaged into the tumor microenvironment by activating various cytokines^42^. Previous studies reported that different types of B cells play different roles in tumor microenvironment. B cells in the tertiary lymphoid structure play an anti-tumor immune role, while regulatory B cells play a role in promoting tumor immune escape^43^. As the most important antigen-presenting cell (APC) in the body, dendritic cells (DC_S_) play a vital role in activating the immune system and leading to T cell differentiation. Furthermore, dendritic cells vaccines have a positive effect on the prognosis of HCC^44^.

In recent years, immunotherapy strategies including immune checkpoint inhibitors have significantly changed the treatment outcomes of HCC and have become a promising approach for cancer treatment^20^, ^45^. Therefore, we further analyzed the relationship between MCM3 and immune checkpoint related genes. Interestingly, our results confirmed that MCM3 was positively correlated with immune checkpoints such as CTLA4, HAVCR2, PDCD1, TIGIT, CD274, and LAG3. Recent studies have shown that immune checkpoint inhibitors such as anti-PD-1, anti-PD-L1, and anti-CTLA-4 antibodies have potential therapeutic effects on advanced HCC^46^. These findings confirm that MCM3 may provide predictive function for immune checkpoint blocking effectors of HCC and play an important role in the immune process. Our study offers some valuable insights but also has certain limitations to consider. The tumor microenvironment, immune system, and physiological characteristics of a single mouse model are specific and may not fully represent the biological traits of other mouse strains or humans. Future research should use multiple mouse models to study tumor growth *in vivo* to more accurately reflect the mechanisms of tumor growth and treatment response across different biological systems. This approach would help validate the conclusions of our study and improve the generalizability of the results.

## Conclusions

In conclusion, our study confirms that MCM3 is highly expressed in HCC and is associated with poor prognosis in HCC patients. The potential oncogenic function of MCM3 in HCC was indicated by GO and KEGG analysis and verified by *in vitro* and *in vivo* experiments. In addition, MCM3 is associated not only with immune cell infiltration but also with immune checkpoint gene expression. Taken together, MCM3 has great potential as a treatment and prognostic marker for HCC.

## Figures and Tables

**Table 1:** Primer sequences.

Name	Sequence (5'-3')
MCM3 forward primer	CTTCTAATAGGAGACCCATCCG
	
MCM3 reverse primer	AATTCATCAATGCAAACCACGC
	
GAPDH forward primer	CATGTTCCAATATGATTCCAC
	
GAPDH reverse primer	CCTGGAAGATGGTGATG

**Table 2:** The clinicopathologic parameters data of HCC patient.

Characteristics	MCM3 expression levels		χ2	P
	Low (n=171)	High (n=171)		
Gender			3.312	0.069
Male	120(70.2%)	104(60.8%)		
Female	51(29.8%)	67(39.2%)		
Age			1.104	0.293
≤50	33(19.3%)	41(24.0%)		
>50	138(80.7%)	130(76.0%)		
T classification			11.049	0.011
T1	100(58.5%)	73(42.7%)		
T2	40(23.4%)	45(26.3%)		
T3	26(15.2%)	48(28.1%)		
T4	5(2.9%)	5(2.9%)		
M classification			4.675	0.031
M0	122(71.3%)	139(81.3%)		
M1	49(28.7%)	32(18.7%)		
Tumor stage			12.984	0.005
I	99(57.9%)	72(42.1%)		
II	40(23.4%)	44(25.7%)		
III	29(17.0%)	54(31.6%)		
IV	3(1.8%)	1(0.6%)		

**Table 3:** Cox proportional-hazard regression analysis for OS.

Characteristics	Univariable			Multivariable		
	HR	95% CI	P	HR	95% CI	P
MCM3	1.668	1.151-2.418	0.007	1.601	1.098-2.335	0.014
Age	0.907	0.578-1.425	0.673	-	-	-
Gender	1.130	0.777-1.643	0.522	-	-	-
T classification	1.633	1.345-1.983	<0.001	1.589	0.675-3.743	0.289
N classification	1.305	0.865-1.969	0.204	-	-	-
M classification	1.516	1.008-2.279	0.046	1.679	1.111-2.538	0.014
Tumor stage	1.652	1.348-2.026	<0.001	1.005	0.411-2.456	0.992
